# From optimization to wisdom: Fostering a patient‐centered professional identity

**DOI:** 10.1111/medu.15767

**Published:** 2025-07-09

**Authors:** Diego Lima Ribeiro

**Affiliations:** ^1^ University Medical Center Groningen University of Groningen Groningen The Netherlands; ^2^ University of Campinas Campinas Brazil

## Abstract

Prescribing is not just a clinical task but a space where students integrate clinical reasoning, moral judgment, and emotional regulation, making it a powerful site for identity formation and transformative learning

The recently published realist review on teaching person‐centred care through Medicines Optimization (MO)^1^ offers a timely and welcome contribution to the field. It explores how medical educators can teach medication‐related decisions (including prescribing and deprescribing) as both technical skills and patient‐centred practices. The authors advocate for an approach that prioritizes thoughtful clinical reasoning rooted in person‐centred care, rather than procedural competence alone.

This commentary extends that perspective by arguing that prescribing is not just a routine, automated task. It is a critical point where clinical reasoning, moral judgement and emotional regulation converge, challenging students to integrate these dimensions in real‐time decisions. Prescribing compels learners to navigate uncertainty, conflicting values and the responsibilities of patient‐centred care and in doing so, serves as an entry point into their professional identity formation. To foster this development, we must go beyond teaching pharmacology or prescribing guidelines. As medical educators, we must cultivate pedagogies that support students as cognitive, moral and emotional agents.

Prescribing compels learners to navigate uncertainty, conflicting values and the responsibilities of patient‐centred care.

To begin, consider the cognitive dimension of prescribing. Before a prescription is written, students must engage in clinical reasoning and reach a diagnosis. Clinical reasoning is not just memorizing guidelines or following a diagnostic checklist.^2^ Rather, it is a dynamic process of pattern recognition and analytical thinking that enables physicians to navigate clinical uncertainty toward a reasoned diagnosis. Students soon realize that foundational knowledge—while necessary—is insufficient. They must interpret the signs and symptoms in complex, real‐world contexts, often while trying to avoid cognitive bias. Clinical reasoning demands the constant integration of prior knowledge with new evidence, as well as the ability to revisit and reassess assumptions. For example, imagine John, a 60‐year‐old man presenting with acute chest pain. The student must distinguish among a myocardial infarction, aortic dissection or a panic attack. After arriving at a diagnosis, the complexity deepens: Treatment decisions must balance clinical evidence with contextual factors. If thrombolytics are indicated, the risks must be weighed in light of John's specific case. At this point, a second dimension, which runs parallel to and transcends the clinical aspects of care arises—moral judgement.

Clinical reasoning demands the constant integration of prior knowledge with new evidence, as well as the ability to revisit and reassess assumptions.

From cognitive complexity emerges the question: What is the right thing to do for this person? Moral judgement begins with intuitive responses—gut feelings about what seems right or wrong—can shift into deliberate moral reasoning when emotionally charged situations expose value conflicts.^3^ In the case of John, diagnosed with ST‐elevation myocardial infarction (STEMI) in a setting without percutaneous coronary intervention, the student must decide whether to prescribe thrombolytics. How should they communicate the bleeding risk? How can they align clinical urgency with the patient's understanding of acceptable risk? These are not communication challenges; they are moral negotiations requiring humility, sensitivity to the patient's perspective and responsibility. The student must act within their limits while recognizing the patient's central role in decision‐making. Thus, the challenge for students is not only to reason well but also to remain morally present in a space where values may collide and certainty is absent. Prescribing, in this sense, becomes not just a clinical task but a moral endeavour. When we, as educators, recognize morally complex decisions as tensions to explore rather than puzzles to be solved, we invite students into reflective practice. With appropriate support, these moments can foster professional identity formation.^4^ Without it, the resulting tension can crystallize into dissonance, distress or quiet detachment.

Prescribing, in this sense, becomes not just a clinical task but a moral endeavour.

Intertwined with clinical reasoning and moral judgement is the emotional dimension. Prescribing is emotionally charged, particularly in high‐stakes or uncertain situations. In John's case, the student often faces insecurity (e.g. unsure whether EKG truly confirms a STEMI), pressure (e.g. time to thrombolytic therapy is crucial) and the possibility of harm (e.g. risk of catastrophic bleeding). Fear of harming the patient, guilt over uncertain calls and anxiety about how others might judge their competence may surface, yet students often feel compelled to appear confident. They may suppress these feelings (surface acting) or attempt to embody expected emotions (deep acting), leading to dissonance. Over time, such emotional labour can result in detachment and compassionate fatigue, distancing learners from patient‐centred care.^5^ But when we make space to name, explore and reflect upon emotional reactions, these moments may become educationally generative. Emotions can serve as signposts of meaning, not threats to professionalism.^6^ In this light, the emotional presence is not peripheral—it is central to the doctor one wants to become.

Emotions can serve as signposts of meaning, not threats to professionalism.

Prescribing brings together clinical reasoning, moral judgement and emotional regulation—not as separate domains, but as interwoven threads in clinical action. When students engage all three dimensions in the face of real uncertainty, these experiences can become a powerful trigger of transformative learning. The original article highlights transformative learning as a product of curricular alignment and interprofessional exposure. While these structural supports matter, we argue that transformation arises from encounters with uncertainty: When students must act despite doubt, question core beliefs or feel dissonance between what they think, feel and are expected to do. These crucial moments invite a shift from absorbing knowledge to becoming the kind of doctor one aspires to be. Supporting this shift calls for medical educators who recognize prescribing as a site of phronesis—practical wisdom.^7^ Phronesis, in Aristotelian terms, is the art of making practical, wise decisions in the face of moral complexity and practical uncertainty. It is cultivated not by standardization but through mentorship, trust relationship and reflective dialogue. As medical educators, we foster phronesis when we share our own doubts in clinical reasoning, acknowledge our struggles with moral judgement and talk honestly about the emotions that arise in patient care. In doing so, we may help students see that being a good doctor involves not just knowing what to do but also how to act with care and purpose.

Prescribing, then, offers more than a technical challenge; it offers a window into who students are becoming. If we teach it as a practice of formation—not only through systems but also through dialogical mentorship and emotional presence—we may form not just competent prescribers but responsible, compassionate and truly person‐centred physicians.

This commentary joins the realist review in affirming the importance of curricular and interprofessional design. At the same time, we also invite our fellow medical educators to consider a complementary question: How might we teach prescribing not only as a skill but also as a space for reflection on the judgements we make and the emotions we carry in clinical care? Such a shift calls for reflective mentorship, emotional honesty and the courage to engage complexity rather than bypass it.

## AUTHOR CONTRIBUTIONS

**Diego Lima Ribeiro:** Writing – original draft.
